# Characteristics of Recycled Polypropylene Fibers as an Addition to Concrete Fabrication Based on Portland Cement

**DOI:** 10.3390/ma13081827

**Published:** 2020-04-13

**Authors:** Marcin Małek, Mateusz Jackowski, Waldemar Łasica, Marta Kadela

**Affiliations:** 1 Faculty of Civil Engineering and Geodesy, Military University of Technology in Warsaw, ul. Gen. Sylwestra Kaliskiego 2, 01-476 Warsaw, Poland; marcin.malek@wat.edu.pl (M.M.); mateusz.jackowski@wat.edu.pl (M.J.); waldemar.lasica@wat.edu.pl (W.Ł.); 2 Building Research Institute (ITB), ul. Filtrowa 1, 00-611 Warsaw, Poland

**Keywords:** packaging waste, plastic waste, macro-polymeric fiber, recycling, eco-efficient concrete, fiber-reinforced concrete, mix modification, mechanical properties, compressive strength, flexural strength, split tensile strength

## Abstract

High-performance concrete has low tensile strength and brittle failure. In order to improve these properties of unreinforced concrete, the effects of adding recycled polypropylene fibers on the mechanical properties of concrete were investigated. The polypropylene fibers used were made from recycled plastic packaging for environmental reasons (long degradation time). The compressive, flexural and split tensile strengths after 1, 7, 14 and 28 days were tested. Moreover, the initial and final binding times were determined. This experimental work has included three different contents (0.5, 1.0 and 1.5 wt.% of cement) for two types of recycled polypropylene fibers. The addition of fibers improves the properties of concrete. The highest values of mechanical properties were obtained for concrete with 1.0% of polypropylene fibers for each type of fiber. The obtained effect of an increase in mechanical properties with the addition of recycled fibers compared to unreinforced concrete is unexpected and unparalleled for polypropylene fiber-reinforced concrete (69.7% and 39.4% increase in compressive strength for green polypropylene fiber (PPG) and white polypropylene fiber (PPW) respectively, 276.0% and 162.4% increase in flexural strength for PPG and PPW respectively, and 269.4% and 254.2% increase in split tensile strength for PPG and PPW respectively).

## 1. Introduction

**Figure 1:**
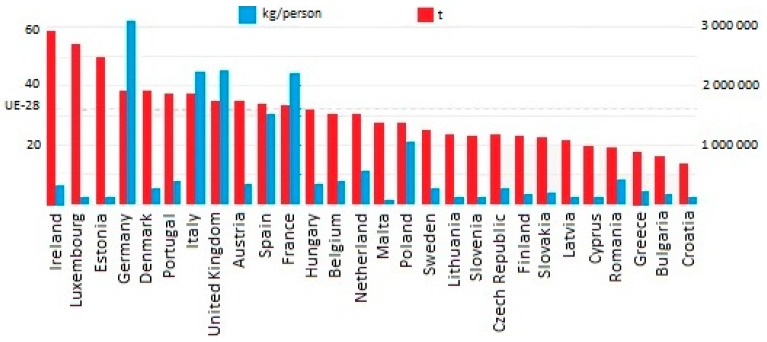
The amount of waste plastic packaging in Europe in 2016 [1,20].

Due to high demand, the global production of fossil-based plastics has grown from 1.5 million tons in 1950 to 288 million tons in 2012 and 322 million tons in 2015. It can be assumed that the upwards trend will continue [1,2,3,4,5]. There are several factors that contribute to the rapid growth of plastics consumption, such as low density, fabrication capabilities, long life, lightness and low cost of production [6]. In addition, plastic is non-corrosive, soft, flexible, not easily damaged, and with high heat and electrical insulation features [7]. For these reasons, plastic has been used widely in the packaging, preservation and distribution of food, and in the housing industry among other uses. For example, the packaging of food accounts for 40% of global plastic consumption (Figure 1) [8]. Taking also into account that plastic is resistant to acids and detergents makes it the safest, and at the same time easiest to produce and cheapest solution for packaging in the food industry. 

The wide application of plastics in most daily activities increases the volume of plastic waste [9]. Approximately half of plastic products are single-use, which causes the generation of different sorts of plastic waste, needing hundreds of years to degrade [8]. This leads to severe environmental concerns, such as human health hazards, effects on animal life, water (between 8 and 24 tons of plastic waste enter the oceans each minute [10,11]) and air pollution (about 400 million tons of CO_2_ [12]) [13], soil impurities [14] and other concerns [15,16,17,18]. Therefore, plastic waste is one of the five priority areas in EU action plan for a circular economy [19].

An effective way to improve this condition is the management of polymer materials in various branches of the economy. Nowadays, large amounts of plastic waste are used in the construction industry. Concrete plays an important role in the process of recycling waste materials [21,22], especially plastic waste, in construction. Plastic waste can be used in concrete as an aggregate (aggregate in concrete or asphalt concrete, synthetic aggregate or as a binder in concrete by melting), resin in polymer concrete, synthetic agent or powder and as synthetic fibers [23,24,25]. According to Czarnecki [26], replacing virgin polymer fibers with recycled waste plastic fibers seems to be becoming a standard development. The properties of recycled fibers can be higher than the properties of virgin fiber and they depend on the manufacturing method, percentage composition and other factors [23].

Traditionally, fibers are used in concrete to improve its post-cracking performance by bridging cracks and preventing the initiation and propagation of shrinkage cracks [27,28,29]. The mechanism of energy absorption and crack control of fiber in concrete was presented in [30,31,32]. By applying polypropylene fibers to the mix, higher tensile strength of the concrete is obtained [33,34,35,36,37]. The effect of polypropylene fibers on the compressive strength of concrete has been discussed in many works. For example, the influence of the addition of polypropylene fibers on the compressive strength of concrete compared to the compressive strength of steel fiber-reinforced concrete was presented in [38,39]. These issues for lightweight concrete were considered in [40], and for high-performance concrete in [41,42,43]. The decrease or increase in compressive strength with the addition of polypropylene fibers was observed. Eidan et al. [44] obtained an almost 8% lower compressive strength for fiber-reinforced concrete compared to samples without fibers. The authors used only one type of polypropylene fibers with a short length (6 mm and 12 mm) and low tensile strength (300–400 MPa). A few studies [38], however, present the influence of the applied addition of recycled polymer fibers on the increase of compressive strength [45]. Akand et al. [46] present a 1–2% increase in compressive strength obtained by adding polypropylene fibers to the concrete mixture. Around a 2% increase in compressive strength for samples with 0.1 wt.% addition of fibers (length 19 mm, diameter 30 μm and tensile strength around 270 MPa) was achieved by Fu et al. [47]. Serrano et al. [39] increased compressive strength by about 74.3% (for 1.0 wt.% fibers) and 73.4% (for 2.0 wt.%) compared to samples without fibers. The slight reinforcement of concrete (around 4.6%) by adding 1.0 wt.% of polypropylene fibers was indicated by Matar and Assaad [48]. They obtained compressive strength equal to around 62 MPa for w/c ratio 0.38, using polypropylene fibers with 80 μm diameter, 12 mm length and tensile strength of 520 MPa. Matar and Assaad have shown that the percentage increase of compressive strength independently from the used w/c ratio (in the article we tested w/c ratio equal to 0.38 and 0.5). Similar increase in compressive strength was observed by Singh [49]: 4.56% with the addition of 0.15% (by volume) of polypropylene fibers (*f_c_* = 38.10 MPa). Tough fibers with 18 μm diameter, 9 mm length, and aspect ratio 500, of 0.91 g/cm^3^ and with low tensile strength (the author does not give the exact value) were used. Moreover, ordinary Portland cement of Grade 43 was used. A percentage increase in compressive strength ranging from 4 to 12% with the addition of 0 to 3% of fibers constructed of plastic waste was determined by Alsadey and Salem [50].

Li et al. [41] obtained a 6.8% higher compressive strength for modified samples with the addition of 3 kg/m^3^ polypropylene fibers than unmodified samples. Similar results were obtained by Qin et al. [45] (6.2% increase compared to samples without fibers). The authors proved that even the small addition of fibers can have higher compressive strength (0.37 wt.% by mass of cement). Fallah and Nematzadeh [51] used different percentage compositions of concrete mix and smaller amounts of polypropylene fibers (0.1, 0.2, 0.3, 0.4 and 0.5 wt.%). They obtained compressive strength equal to 65.6 MPa for the mix with 0.1% fiber 12 mm length, 20 μm diameter and tensile strength around 350 MPa. This value is around 11.5% higher than in samples without fibers. Hiremath and Yaragal obtained much higher compressive strength in concrete with 0.1 wt.% polypropylene fibers, equal to 105 MPa. Such high value is probably the result of a large quantity of cement (900 kg/m^3^) and the lower grain of aggregates: quartz powder (grain size 10–45 μm) and silica sand (grain size 150 ÷ 600 mm). The maximum value of compressive strength obtained by Li et al. [41] is 159.7 MPa. They used high-class cement (CEM I 52.5 N) and a higher ratio of cement (almost the same amount of cement as aggregates), which indicated higher mechanical properties. Li et al. [42] obtained compressive strength equal to 147.2 MPa, as they used an almost double quantity of cement, which highly influences compressive strength. The effect of the type of fibers, their properties, such as length, diameter aspect ratio, and their effect on the properties of concrete changes with the dosage [52].

This study aims to contribute to this growing area of research by exploring the influence of the content of polypropylene fiber on the compressive, flexural and split tensile strength of fiber-reinforced concrete. However, unlike other studies, the subject of this research was the fibers from byproducts of recycling plastic packaging. Two types of recycled polypropylene fibers (green polypropylene fiber (PPG) and white polypropylene fiber (PPW)) were investigated. The fibers were made at the same process of recycling, but PPW were produced using an additional extruder applying a structure to their surface. 

Whereby, the findings of the study make an important contribution towards an effective and ecological solution of utilizing plastic waste (packaging waste) in concrete. 

## 2. Materials

### 2.1. Specimen Preparation

The materials used in this study were Portland cement, water and a polycarboxylate deflocculant used as a hardening admixture. The industrial Portland cement was CEM I 42.5R, according to PN-EN 197-1:2011. Its chemical composition and physical properties, measured as per PN EN 196-6:2011, is given in Table 1. The compressive strength of cement was determined according to PN EN 196-1:2016-07 (Table 2). Tap water was used in all experiments.

**Figure 2:**
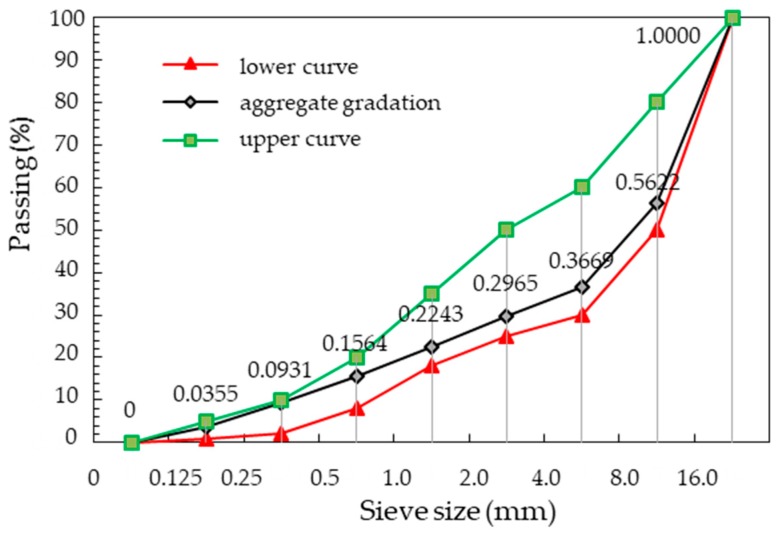
Gradation of quartz sand.

As a filler, quartz sand with fraction of 0.125–0.250 mm, 0.250–0.500 mm and 0.500–1.000 mm was used (Figure 2). The filler is heterogeneous (grain size index *C_U_* = 4.8 and *C_C_* = 1.9).

**Figure 3:**
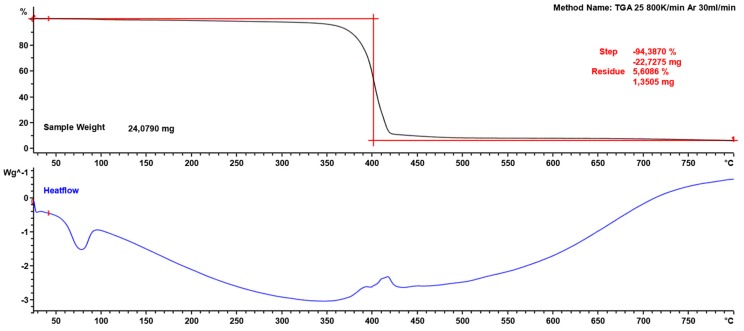
Thermogravimetric analysis of admixture.

In order to increase the workability of the mix, chemical admixtures were added to fiber-reinforced concrete mixes. A chlorine-free, low-alkaline liquid chemical admixture based on an aqueous solution of modified polycarboxylic ethers (melamine and silanes/siloxanes) was used (Table 3). The polymer admixture was added to reduce significantly the amount of water (maintaining water-cement ratio w/c at 0.26), limit the phenomenon of “bleeding” and delay the setting of the cement paste. Figure 3 presents the thermogravimetric analysis of the admixture.

### 2.2. Polypropylene Fibers

**Figure 4:**
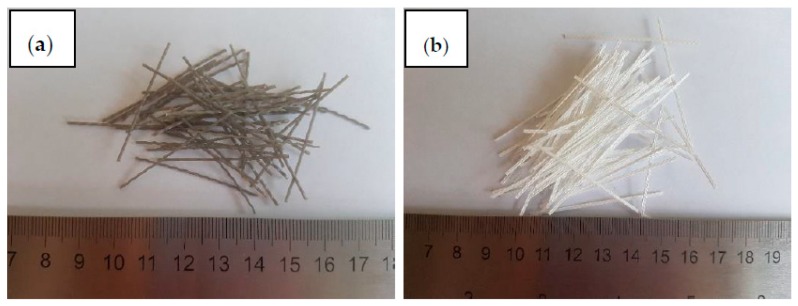
Polypropylene recycled fibers: (**a**) green polypropylene fiber (PPG) and (**b**) white polypropylene fiber (PPW).

Two polypropylene fibers from waste materials were used (Figure 4). Both fibers were made from the same granulate product of polypropylene-plastic packaging-and they are chemically identical. The first (green polypropylene fiber—PPG) is made from the cutting process of waste material, while the second fiber (white polypropylene fiber—PPW) was modified into the extruder to increase its adhesion to the cementitious mix.

Polypropylene fibers of about 31 mm length (30.9 ± 0.5 mm for PPG and 31.2 ± 0.5 mm for PPW) and 1.000 microns in diameter were used. The tensile strength of fibers is about 520 MPa. Table 4 presents the basic properties of the tested fibers. Fiber ratios of (0%), (0.5%), (1.0%) per (1.5%) of weight of cement have been used. 

### 2.3. Mix Composition

Three different types of mortar mixes (one without fibers, and the rest with different content of fibers) were produced. The polypropylene fiber content was 0.5, 1.0 and 1.5 wt.% (by mass of the cement), marked in the article as 0.5PPG or 0.5PPW, 1.0PPG or 1.0PPW, and 1.5PPG or 1.5 PPW depending on the type of fiber (green—PPG or white—PPW). In total, seven different mixes were produced to investigate the effect of polypropylene fiber content on the properties of cementitious mix (see Table 5). A constant w/c = 0.3 ratio was used for all mixes. The admixture content was 3.0% of the mass of cement, which is consistent with [33,54].

### 2.4. Mix Production

The fibers were added to the cementitious mixture, after mixing other components for 2 min. The whole mixing time lasted for a maximum of 5 min. All samples were obtained in laboratory conditions (21 °C temperature and 50% humidity). The samples were stored in water according to PN-EN 12390-2:2019.

## 3. Methodology

### 3.1. Test on Mix

The initial and final setting times and the drop cone test were determined. The initial and final setting times were measured with the Vicat apparatus (Poznań, Poland).

The workability of fresh concrete was determined using a slump cone according to PN-EN 12350-2:2019. The presented in article values are average values of five measured specimens for each mix.

### 3.2. Test on Hardened Concrete

The mechanical properties of the mortar (compressive strength and flexural strength) were determined. The samples were tested using a Zwick machine (force range 0–5000 kN, Ulm, Germany). Compressive strength was measured on samples 100 mm × 100 mm × 100 mm, according to PN-EN 12390-3:2019 + AC:2012.

Flexural strength was tested in a three-point bending set-up with beams 40 mm × 40 mm × 160 mm, according to PN-EN 12390-5:2019. The nominal distance between the supports was 100 mm. The rollers allowed for free horizontal movement. 

Split tensile strength was tested on a cylinder 0.15 m in diameter and 0.30 m in height, according to PN-EN 12390-6:2011.

The above tests were carried out after 1, 7, 14 and 28 days of curing process to evaluate the behavior of the mix with two types of polypropylene fibers in time. The presented values are the average values of three samples for each mix.

## 4. Results and Discussion

### 4.1. The Initial and Final Setting Time

**Figure 5:**
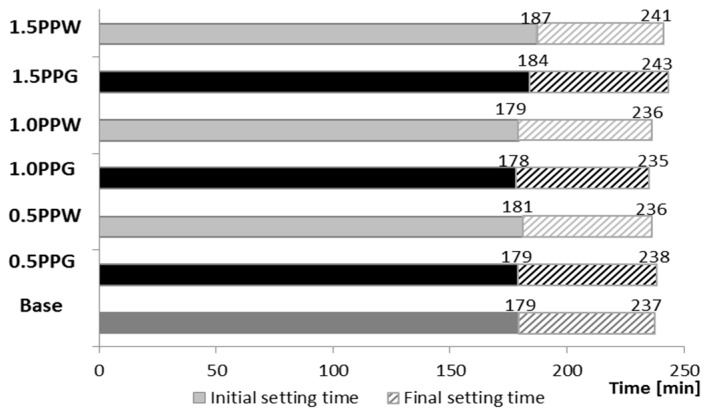
Distribution of initial and final setting times of tested mixes: PPG marked in black, PPW marked in light grey, base mix (no fibers) marked in dark grey.

The distribution of the initial and final setting times for the mixes with different content of polypropylene fibers for two different types of fiber are presented in Figure 5, against the results obtained for the base mix (no fibers), marked in dark grey in Figure 5. In each case, higher results were obtained for PPW, but the difference is within the error limit of the measurement method. As such, it may be accepted that no effect of fiber addition on setting time was observed. The initial and final setting times are similar for all mixes (except for a mixture with 1.5% fiber) and are equal to 179 ± 1 and 236 ± 1 min respectively. Similar initial setting time (130 min) was presented in [54], but the final setting time is different. Das et al. received a 62.5% higher value of final setting time (390 min). Li et al. [35] received a very similar value of final setting time (255 min), but about 37% times lower value of initial setting time (115 min). This is due to the different mix compositions. In the first case, a different type of cement (Original Portland Cement) was used which indicated the discrepancy of the results. In the second case, there is no information about the used superplasticizer, which can affect the initial and final setting times. The influence of the mix composition on final setting time was presented by Matar and Assaad [48]. Samples with the highest mechanical properties (with 1.0 wt.% polypropylene fiber addition) showed 18 h 5 min final setting time. This value is 4.6 times higher than the one obtained by the authors of this paper, but different binders were used (70% Portland cement ASTM C150 Type I, 25% blast furnace slag ASTM C989 Grade 100 and 5% silica fume) and 0.38 w/c ratio.

**Figure 6:**
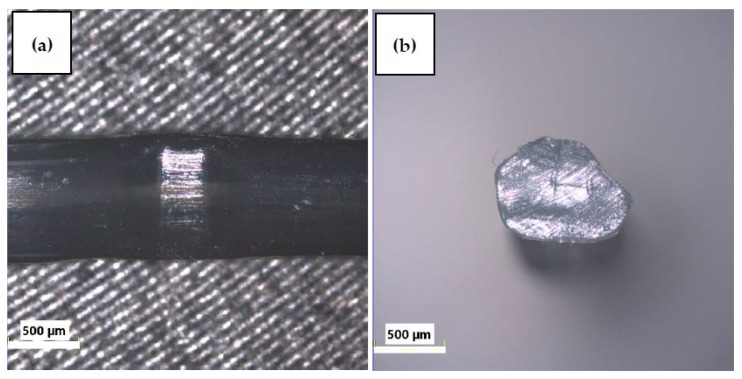
Light microscope images of PPG (Olympus): (**a**) surface, (**b**) cross-section [55].

**Figure 7:**
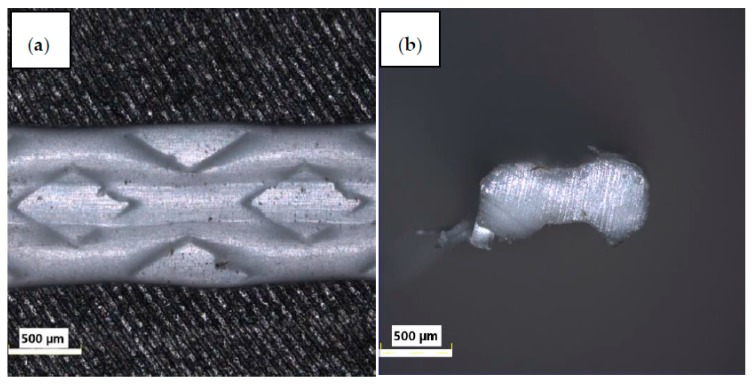
Light microscope images of PPW (Olympus): (**a**) surface, (**b**) cross-section [55].

**Figure 8:**
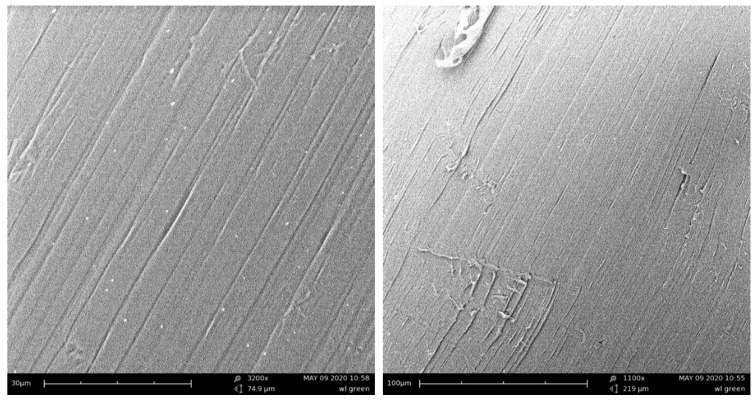
Scanning electron microscope (SEM) images of PPG.

**Figure 9:**
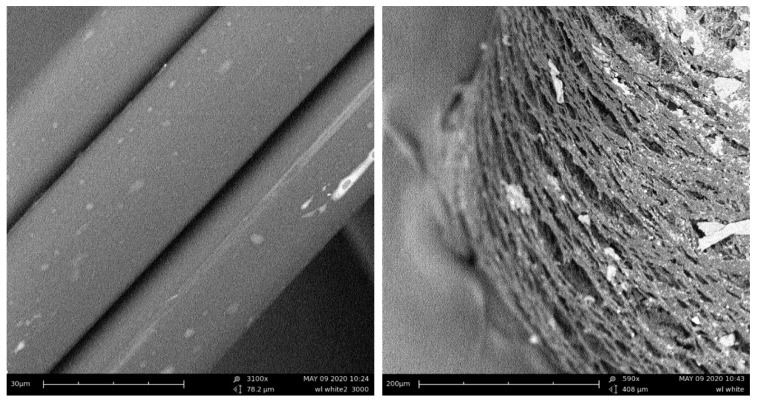
Scanning electron microscope (SEM) images of PPW.

The influence of the fibers’ external structure is not apparent in tests. The surface of PPG is smooth, while the surface of PPW is irregular, which is due to the modification process of PPW (Figure 6, Figure 7, Figure 8 and Figure 9). Additionally, the different structures of the fibers did not affect the mixing process. During the mixing, no process of agglomeration formation was observed. The fibers did not float to the surface nor sink to the bottom in the fresh concrete. Both fibers were mixed smoothly with mortar mix. These behaviors can be the result of chemical admixture.

**Figure 10:**
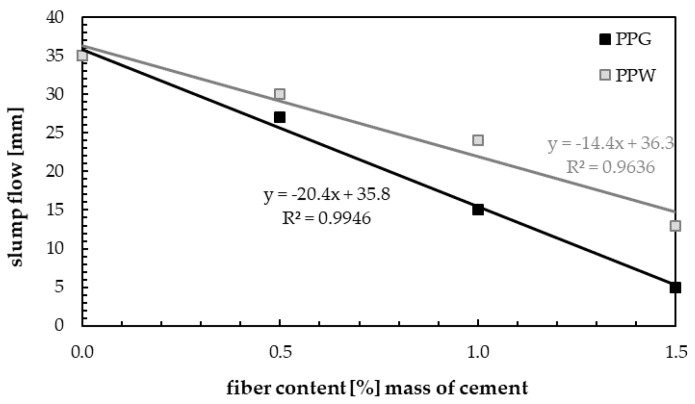
Slump test results.

The result of slump flow (SF) test were shown in Figure 10. The addition of PP fibers to plain concrete reduces its workability. At the same trend was observed by other scientists [56,57] and with the addition of 0.5, 1.0 and 1.5 wt.% of fibers, the decrease increase in slump was 22.9%, 57.1% and 85.7% for PPG compared to plain concrete, while 14.3%, 31.4% and 62.9% for PPW. All mixes were within slump flow class SF1. A larger decrease in slump was observed for concrete with addition of PPG fiber. Similar decreases in slump (84.2% and 88.2%) with increasing fibers content were obtained by Mohammadhosseini and Tahir [14] for the two types of plastic fibers analyzed. Additionally, Bayasi and Zeng [58] determined 88.4% of decrease in slump for fresh concrete with polypropylene fibers compared to mix of plain concrete.

### 4.2. Compressive Strength

**Figure 11:**
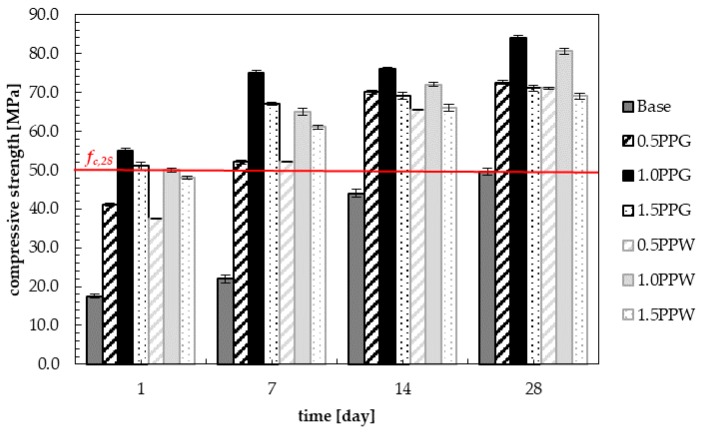
Compressive strength of samples with PPG and PPW during the curing process.

The results of compressive strength for samples with the addition of 0.5, 1.0 and 1.5 wt.% of fibers (PPG or PPW) were shown in Figure 11, along with the results of hardening samples with green (PPG) and white polypropylene fibers (PPW) marked in black and light grey respectively. The results were compared with the base mix (without fibers) marked in dark grey. The compressive strength increased with the addition of fibers for all samples of reinforced concrete. The same phenomenon was observed by Afroughsabet and Ozbakkaloglu [38], and Fallah and Nematzadeh [51]. All obtained in this study 28 days compressive strengths were above 60 MPa (from 69.0 to 84.0 MPa for different content of two types fibers, while compressive strength of plain concrete was equal 49.50 MPa). Thus, the concrete with recycled polypropylene fibers met the requirements for high-strength concrete [59,60]. 

The highest values of compressive strength during the entire curing process were obtained for the samples containing 1.0 wt.% of polypropylene fibers. After one day of curing, the compressive strength for samples with 1.0% content of PPG was three times higher (55.0 MPa) than for the base sample (without fibers). After seven days, it was about 3.5 higher (75.0 MPa) than for the base sample. In the following days (14 and 28 days), the compressive strength of fiber-reinforced concrete was higher by approximately two times compared to unreinforced concrete, and was equal to 76.0 and 84.0 MPa respectively. For concrete with white polypropylene fibers (PPW), the values were about 5 to 10% lower than for samples with PPG and they were equal to 50.0 MPa after 1 day, 65.0 MPa after 7 days, 72.0 MPa after 14 days and 80.5 MPa after 28 days. It can be observed that with the addition of 0.5 wt.% of fiber, in 1, 7, 14 and 28 days compressive strength was increased compared to normal concrete by 134.3%, 136.4%, 59.1% and 46.5% for PPG, and 114.3%, 136.4%, 48.9% and 43.4% for PPW. With the addition of 1.0 wt.% of fibers, the increase in strength compared to unreinforced concrete is 214.3%, 240.9%, 72.7% and 69.7% for PPG, and 185.7%, 195.5%, 63.6% and 62.6% for PPW. With the addition of 1.5 wt.% of fibers, this increase is 191.4%, 204.6%, 56.8% and 43.4% for PPG, and 174.3%, 177.3%, 50.0% and 39.4% for PPW.

Each time, higher values of compressive strength were observed for green polypropylene fibers (PPG), which were slightly longer and thinner (Table 4) and with smoother surfaces (Figure 6 and Figure 8), than for PPW fiber with the surface structure applied (Figure 7 and Figure 9). The observed phenomenon is different from the results of other studies [61] and provides confirmation of observation [62]. This is due to the addition of fibers, the way the concrete sample was destroyed, and at the same time the way the value of the strength of the concrete was changing. The concrete composite did not get destroyed after cracking but was further deformed while still maintaining load bearing capacity (post-cracking performance) [63,64]. The fibers are able to bridge the cracks and transfer stress across the cracks [65]. The mechanism of energy absorption and crack control of fiber in the concrete was presented in [32]. The fiber reinforced concrete is destroyed in the moment when the fiber slides out of the matrix or breaks (in second case the load is redistributed to the other fibers [66]). Thus, the method of damage of the fiber reinforced concrete mostly depends on the strength of the materials and the adhesion of the fibers to the matrix. The phenomena of the mechanical engagement between the fiber, aggregate and concrete matrix particles or the phenomenon of fiber surface adhesion to fiber occur [67,68,69].

**Figure 12:**
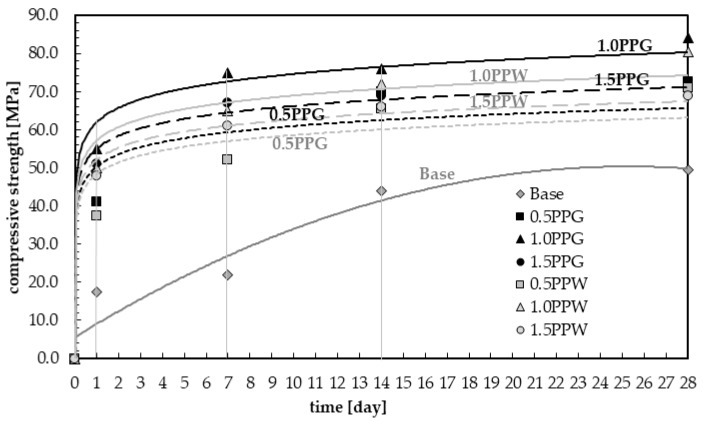
Compressive strength of samples in the function of curing process time with marked times analyzed in this study.

For all analyzed samples with different fiber content the required compressive strength *f_c,28_* was achieved earlier than for unreinforced concrete (without fibers), between 1 and 7 days. Figure 11 and Figure 12 show that in the first day, this requirement was met for concrete with 1.0 wt.% fibers (PPG and PPW) and with 1.5 wt.% PPG. Then, increase in strength was slow. The relationships between the compressive strength and curing time for fiber reinforced concrete can be found by using Equations (1)–(6). Equations (1), (2) and (3) were determined for concrete with 0.5, 1.0 and 1.5 wt.% addition of PPG fibers, marked 0.5PPG, 1.0PPG and 1.5PPG in Figure 12. While the empirical Equations for concrete with 0.5, 1.0 and 1.5 wt.% addition of PPW fibers are as mentioned in Equations (4), (5) and (6) and marked 0.5PPG, 1.0PPG and 1.5PPG in Figure 12, respectively. The coefficients of determined *R*^2^ for Equations (1)–(6) were 0.9274, 0.985, 0.9931, 0.9252, 0.9754 and 0.9929, respectively. The relationship for plain concrete, marked as base, was the background in Figure 12. The empirical formula was found as expressed in Equation (7) with *R*^2^ = 0.9185.

(1) $$f_{c}=4.5902\cdot \ln\left(t\right)+50.401$$

(2) $$f_{c}=5.4999\cdot \ln\left(t\right)+61.955$$

(3) $$f_{c}=4.8387\cdot \ln\left(t\right)+55.080$$

(4) $$f_{c}=4.4340\cdot \ln\left(t\right)+48.389$$

(5) $$f_{c}=5.1188\cdot \ln\left(t\right)+57.181$$

(6) $$f_{c}=4.5995\cdot \ln\left(t\right)+52.108$$

(7) $$f_{c}=-0.0698\cdot t^{2}+3.5337\cdot t+5.6489$$

It can be observed that the nature of the recycled fiber-reinforced concrete curing process is faster than for the reference concrete (without fibers). This allows it to be used in places where high strength is required fast. The faster stabilization in curing time with the addition of polymer was observed by Albano et al. [70]. They replaced sand with polyethylene terephthalate (PET) in concrete.

**Figure 13:**
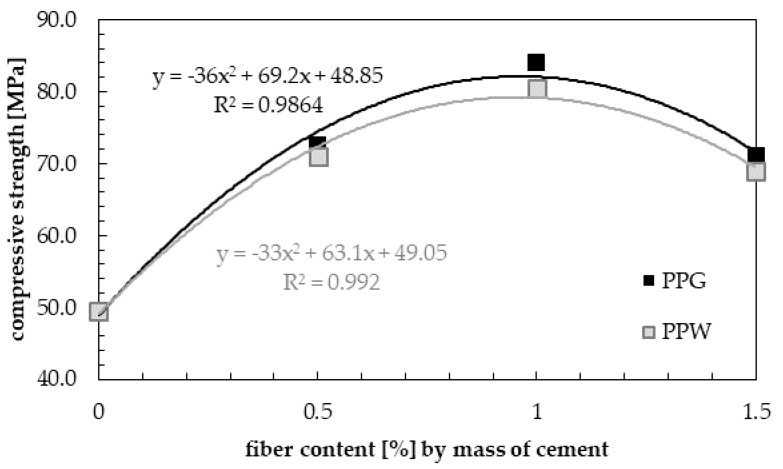
Compressive strength of hardened samples with different content of PPG and PPW.

Figure 13 presents the test results of compressive strength at 28 days. The compressive strength increases with increasing fiber concentration up till 1.0%, above which compressive strength decreased. The 1.0% addition of polypropylene recycled fibers into the concrete showed maximum benefits in compressive strength of hardened samples, regardless of the type of fiber. Other studies [39,46,48] have come to the same conclusion, but the values (*f_c_* = 84.00 MPa for PPG and 80.50 MPa for PPW) and/or an increase of compressive strength (69.7% for PPG and 63.6% for PPW) of fiber-reinforced concrete compared to normal concrete were lower. Bayasi and Zeng [58] obtained about a 20% increase in compressive strength. Serrano et al. [39] observed a 74.3% increase in compressive strength at low values of compressive strength due to using a lower grade of cement (CEM II/BL 32.5). The resulting increase in compressive strength is more than twice that reported by Dharan and Lal [71]. Higher increase in compressive strength than that presented in this article at high values of compressive strength was obtained for ultra-high performance concrete with steel fibers. An increase up to 117.6% and 113.8% with the addition of 1.0% fiber content with aspect ratio 80 and 65 respectively, and 15% silica fume compared to the control specimen were obtained by Köksal et al. [72].

This effect of the addition of recycled fibers is unexpected and unparalleled in the results of other works. The properties of fiber-reinforced concrete depend on the type of fiber, its size and properties, aspect ratio, distribution of fibers and other features [73]. The used polypropylene fibers are tough and characterized by aspect ratios of 33 and 24 for PPG and PPW respectively. This can have an effect on the obtained results. Moreover, higher values were observed for longer fibers, so this is compatible with the theory that compressive strength is improved with an increase in fiber length [74].

### 4.3. Flexural Strength

**Figure 14:**
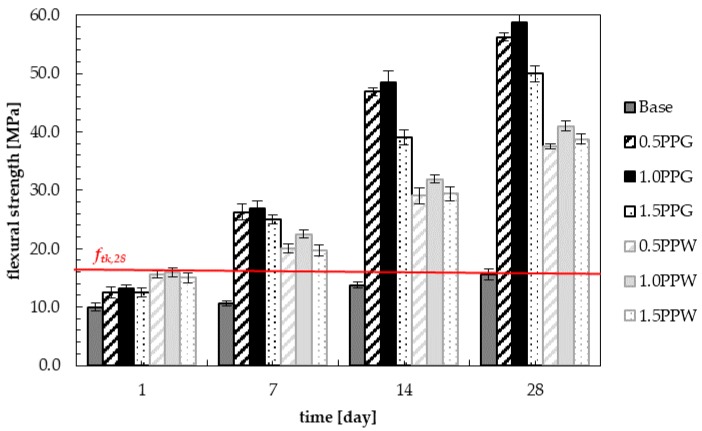
Flexural strength of samples with PPG and PPW during curing.

Figure 14 shows the results of the flexure test of samples with green (PPG) and white polypropylene fibers (PPW). The highest values of flexural strength during the entire curing process were obtained for the samples containing 1.0 wt.% of polypropylene fibers. After one day of the curing process, samples with white polypropylene fibers (PPW) showed higher values of flexural strength (15.9 MPa) and were equal to the values after 28 days of flexural strength of normal concrete (*f_tk,28_ =* 15.6 MPa). In the following days, flexural strengths of all samples of fiber-reinforced concrete were higher than 28 days of flexural strengths of base concrete. The highest values of 7, 14 and 28 days of flexural strength were obtained for the samples with the addition of 1.0 wt.% PPG. They were equal to 26.9 MPa for 7 days, 48.4 MPa for 14 days and 58.8 for 28 days. These values are about 2.5, 3.5 and 3.8 times higher than for unreinforced concrete respectively and about 1.2, 1.5 and 1.4 times higher than for samples with the addition of 1.0 wt.% PPW. Mehta and Monteiro indicated that the inclusion of 1.25% steel fiber content caused flexural strength to increase by about two times [62]. 

It can be observed that with the addition of 0.5 wt.% of fiber 1, 7, 14 and 28 days compressive strength was increased compared to normal concrete by 25.0%, 147.1%, 240.9% and 260.0% for PPG, and 56.3%, 88.2%, 111.4% and 140.0% for PPW. With the addition of 1.0 wt.% fibers, the increase in strength compared to the reference concrete is 31.3%, 152.9%, 252.3% and 276.0% for PPG, and 59.4%, 111.8%, 131.8% and 162.4% for PPW. With the addition of 1.5 wt.% fibers, this increase is 25.0%, 135.3%, 184.1% and 220.0% for PPG, and 50.0%, 85.9%, 113.6% and 148.0% for PPW. Mohammadi et al. [75] reported that the inclusion of 1.0%, 1.5% and 2.0% steel fiber content led to an increase in the flexural strength of concrete varying from 28 to 61%, 45 to 95% and 43 to 167% respectively, depending on the weight of the long and short steel fibers in the concrete. Blunt and Ostertag [76] obtained an increase up to 196% in flexural strength relative to the control high-performance concrete using fiber hybridization.

**Figure 15:**
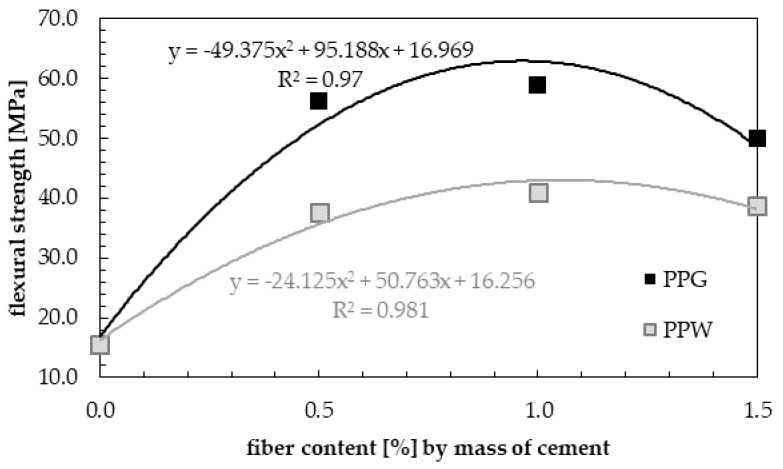
Flexural strength of hardened samples with PPG and PPW.

Flexural strength increased with the increasing percentage of fibers for all samples of reinforced concrete up to a certain fiber content and then decreased. This trend is consistent with the observations of Singh [49] and Dharan and Lal [71]. The 1.0% addition of polypropylene recycled fibers into the concrete showed maximum benefits in flexural strength of hardened samples, regardless of the type of fiber (Figure 15). The same conclusion was determined by Badogiannis et al. [40]. By adding 1.0 wt.% polypropylene fibers, however, they only obtained around a 37% increase in 28 days of flexural strength compared to unreinforced concrete. Badogiannis et al. used longer and thinner fibers (length 52 mm, diameter 46 μm), see Table 4. Rostami et al. [34] used shorter but similar in diameter fibers to Badogiannis et al. (18 mm length, 39.78 μm diameter, weight 12 g and tensile strength 258.05 MPa) and obtained 70.3% increase in flexural strength with the addition of 0.55 wt.% polypropylene fibers in concrete. They also, however, obtained much lower maximum values of flexural strength equal to 6.3 MPa due to using different concrete mixture components and proportions. Other scientists present an increase in flexural strength for polypropylene fiber-reinforced concrete ranging from about 10% [36,38,77] to 35% [71]. An increase in flexural strength can be observed with significant proportions of polypropylene fibers [34,77,78].

### 4.4. Split Tensile Strength

**Figure 16:**
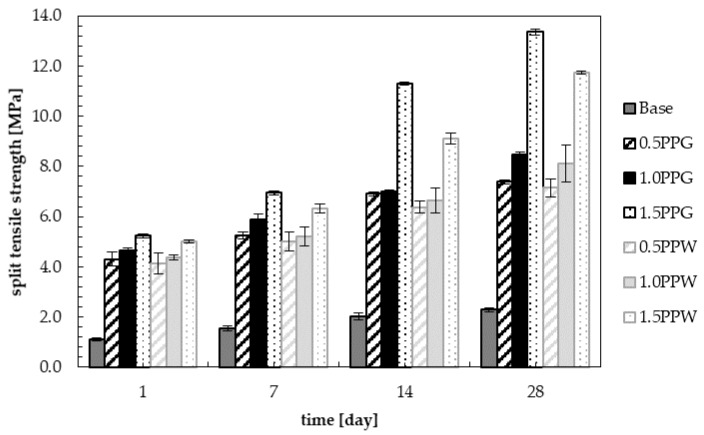
Split tensile strength of samples with PPG and PPW during curing.

The results of split tensile strength for samples with the addition of 0.0%, 0.5%, 1.0% and 1.5 wt.% of fibers (PPG or PPW) are shown in Figure 16. The highest values of split tensile strength during the entire curing process were obtained for the samples with maximum percentage of polypropylene fibers (1.5 wt.% of polypropylene fibers). This is consistent with the observations of Dharan and Lal [71].

**Figure 17:**
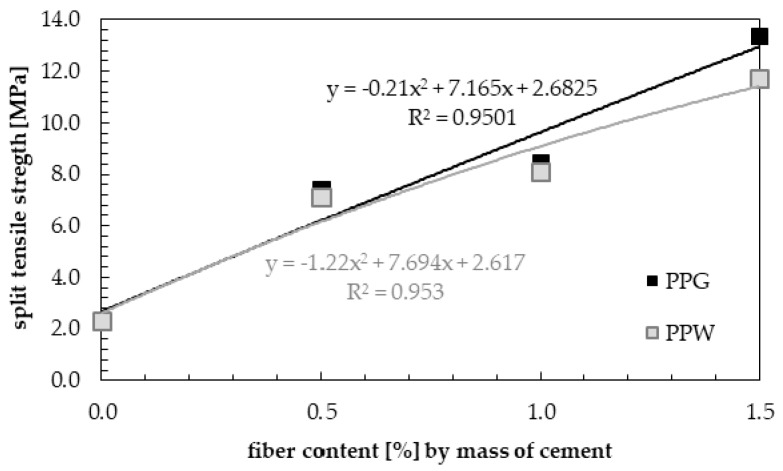
Split tensile strength of hardened samples with PPG and PPW.

It can be observed that with the addition of 0.5 wt.% of fibers, 1, 7, 14 and 28 days split flexure strength were increased compared to normal concrete by 284.8%, 237.2%, 241.6% and 222.7% for PPG, and 268.8%, 221.2%, 215.8% and 211.8% for PPW. With the addition of 1.0 wt.% of fibers, the increase in strength compared to the reference concrete is 315.2%, 276.3%, 246.5% and 269.4% for PPG, and 291.1%, 234.0%, 229.2% and 254.2% for PPW. With the addition of 1.5 wt.% of fibers this increase is 367.0%, 345.5%, 459.4% and 483.0% for PPG, and 347.3%, 305.1%, 351.0% and 412.7% for PPW. Split tensile strength increased with non-linear increasing percentage of fibers for all samples of reinforced concrete, regardless of the type of fiber (Figure 17).

The obtained increase of split tensile strength is a novelty in research on polypropylene fiber-reinforced concrete. Dharan and Lal [71] obtained 25.7% increase in split tensile strength. Raghatate [73] determined 35.2% increase in strength. Moreover, marginal increase in split tensile strength was observed by Kandasamy and Murugesan [79]. Song et al. [80] reported 9.7% increase in split tensile strength. Thus, high values of increase in split tensile strength have been reported for the addition of steel fibers [71,81,82,83,84,85,86,87]. They determined that for low contents (0.5% < Vf ≤ 1.0%) and higher contents (1.0% < Vf ≤ 2.0%) of fibers, improvements were observed in the split tensile strength values, which varied from 15 to 121%, and 40 to 143% respectively. Song and Hwang [88] obtained an increase in split tensile strength ranging from 19.0 to 98.3%, depending on the fiber content (up to 2.0%) compared to the base concrete. According to the results of Wang and Wang [89], the split tensile strength increased by 92.5% with the addition of 2.0% of steel fiber with 32 mm length and an aspect ratio of 50 compared to the control concrete. For the same dosage of steel fibers, but with an aspect ratio of 60, which included 10% silica fume, split tensile strength was increase by 129.9% in tests carried out by Eren and Çelik [90]. Higher increase equal to 159.8% with the addition of 1.5% hooked-end steel fibers was obtained by Wafa and Ashour [91].

## 5. Conclusions

The aim of the study was to assess the possibility of using fiber made from used (recycled) PP packaging as an alternative to traditional fibers used in reinforced concrete. This experimental work has included three different contents (0.5, 1.0 and 1.5 wt.% of cement) for two types of macro-polymeric fibers (green polypropylene fiber PPG with smooth surface and white polypropylene fiber PPW with the surface structure applied). Based on the results of this experimental investigation, the following key conclusions can be drawn:

(1) The decrease in the slump flow with addition of PP fibers was observed, but all mixes were within slump flow class SF1;
(2) The obtained 28 days compressive strengths of reinforced concrete were from 69.0 to 84.0 MPa for different content of fibers, what meet the requirements for high-strength concrete;
(3) The increase in mechanical properties as an effect of the addition of recycled polypropylene fibers compared to unreinforced concrete is unexpected and unparalleled (69.7% and 39.4% increase in compressive strength, 276.0% and 162.4% increase in flexural strength and 269.4% and 254.2% increase in split tensile strength were obtained for PPG and PPW respectively);
(4) The highest values of mechanical properties were obtained for concrete with 1.0 wt.% of polypropylene fibers for each type of fiber;
(5) The higher values for all tested properties were determined for PPG fibers;
(6) The nature of the recycled fiber-reinforced concrete curing process is faster than for the reference concrete (without fibers);
(7) For all fiber-reinforced concrete samples the required compressive strength was achieved earlier than for normal concrete (between 1 and 7 days) which allows it to be used in places where high strength is required fast.

The present work belongs to a wider research project aimed at developing environmentally friendly concrete according to sustainable development and the circular economy rules. Therefore, as part of further research, we plan to carry out tests for other types of cement, other types of plastic waste recycled fibers, and different contents and dimensions of those fibers, with particular emphasis on fatigue tests.

## Figures and Tables

**Table 1:** Chemical composition of cement.

**Compositions**	SiO_2_	Al_2_O_3_	Fe_2_O_3_	CaO	MgO	SO_3_	Na_2_O	K_2_O	Cl
**Unit** **(vol.** **%)**	19.5	4.9	2.9	63.3	1.3	2.8	0.1	0.9	0.05

**Table 2:** Physical properties of cement [53].

Specific Surface Area [m^2^/kg]	Specific Gravity [kg/m^3^]	Compressive Strength after Days [MPa]	
		2 days	28 days
3840	3060	28.0	58.0

**Table 3:** Chemical composition of admixture.

**Compositions**	O	Na	S	K
**Unit** **(vol.** **%)**	77.7	14.9	4.8	2.6

**Table 4:** Properties of the green (PPG) and white polypropylene fibers (PPW) (own research).

Type of Fiber	Average Thickness [μm]	Average Circumference [μm]	Length [mm]
PPG	904.9 ± 10.0	472.2 ± 0.5	29.7–33.2
PPW	1152.5 ± 10.0	538.2 ± 0.5	27.1–32.6

**Table 5:** Mix proportions (1 m^3^).

Mix Symbol	Cement [kg]	Water [kg]	Type of Fiber	Fiber Content [wt.%]	Fiber [kg]
Base mix	550	165	–	0.0	0.0
PPG-0.5			PPG	0.5	2.75
PPG-1.0				1.0	5.50
PPG-1.5				1.5	8.25
PPW-0.5			PPW	0.5	2.75
PPW-1.0				1.0	5.50
PPW-1.5				1.5	8.25
